# Community Awareness on Rabies Prevention and Control in Bicol, Philippines: Pre- and Post-Project Implementation

**DOI:** 10.3390/tropicalmed3010016

**Published:** 2018-02-01

**Authors:** Toni Rose M. Barroga, Ilene S. Basitan, Themis M. Lobete, Rona P. Bernales, Mary Joy N. Gordoncillo, Emelinda L. Lopez, Ronello C. Abila

**Affiliations:** 1 Bureau of Animal Industry-World Organisation for Animal Health Stop Transboundary Animal Diseases and Zoonoses Rabies Project, Visayas Avenue, Diliman, Quezon City 1100, Philippines; 2 College of Veterinary Medicine, Central Bicol State University of Agriculture, Pili, Camarines Sur 4418, Philippines; ilene.basitan@cbsua.edu.ph; 3 Municipal Agriculture Office, Municipal Government of Daraga, Daraga, Albay 4501, Philippines; themislobt@gmail.com; 4 Department of Agriculture Regional Field Office V, Pili, Camarines Sur 4418, Philippines; rona_bernales@yahoo.com; 5 World Organisation for Animal Health Sub Regional Representation for Southeast Asia, Phaya Thai Road, Ratchathewi, Bangkok 10400, Thailand; mgordoncillo@gmail.com (M.J.N.G.); r.abila@oie.int (R.C.A.); 6 Food and Agriculture Organization of the United Nations (UN-FAO), Regional Office for Asia and the Pacific (RAP), Phra Atit Road, Phra Nakorn, Bangkok 10400, Thailand; 7 Animal Health and Welfare Division, Bureau of Animal Industry, Visayas Avenue, Diliman, Quezon City 1100, Philippines; doc_minnie12@yahoo.com

**Keywords:** attitude, knowledge, practice, rabies, community

## Abstract

Rabies is endemic in the Philippines. To support the rabies campaign in the Bicol region at the southeastern part of Luzon, the BAI-OIE Stop Transboundary Animal Diseases and Zoonoses (STANDZ) Rabies project was implemented in the pilot provinces of Camarines Norte, Camarines Sur, Albay, and Masbate. A community awareness survey was conducted with the residents of these provinces to determine their knowledge, attitude, and practices (KAP) on rabies during the start and end of the project. Qualitative, descriptive research was done with a structured KAP questionnaire. Pet owners in the pilot provinces were chosen as respondents. Results showed that respondents know that they can acquire rabies in animals through the bite of a rabid dog (pre-project implementation (PRI): 19.6%, post-project implementation (POI): 38.0%). Vaccination was the top rabies preventive measure (PRI: 61.8%, POI: 92.8%). Biting incidents were noted in some respondents, and observing the dog and killing it immediately were some of the actions taken by bite victims. If a supposed rabid dog was seen, respondents would either: immediately kill the dog (PRI: 20.3%, POI: 13.7%), report it to authorities (PRI: 26.3%, POI: 63.1%), and capture and observe the dog concerned (PRI: 13.5%, POI: 6.0%). Pet owners increased their KAP about rabies prevention and control as compared to the pre-implementation study. However, certain gaps in their KAP need to be given attention; thus continuous education of pet owners must be done.

## 1. Introduction

Rabies is considered a neglected tropical disease and is responsible for claiming the lives of 59,000 humans annually, mainly in developing countries of Asia and Africa [1]. Once clinical symptoms appear, death is inevitable; however, with timely post-exposure prophylaxis (PEP) after exposure to a rabid animal, the disease is preventable [2]. Globally, ninety percent of all rabies-related human deaths are due to dog bites; thus, the conduct of mass dog vaccination (MDV) is seen as the most cost-effective measure in controlling rabies at its source [3,4,5].

In the Philippines, rabies remains endemic with 200–300 human fatalities annually (an average of 2.13 deaths per million) and 635 animal rabies cases (an average of 6.35 rabid dogs per 100,000) since 2012 [6]. In an effort to reduce the impact and ultimately eliminate this disease in humans, the Philippine government has strengthened eradication programs directed towards its source in dogs. Through the passage of the Republic Act No. 9482, otherwise known as the Anti-Rabies Act of 2007, provinces, cities, and municipalities are mandated by this law to ensure that all dogs are properly vaccinated and registered. This also includes strengthening rabies diagnostic laboratories and surveillance, the provision of additional post-exposure prophylaxis (PEP) for animal bite patients, and promoting responsible pet ownership (RPO) [7].

Financial provision to facilitate these activities is a crucial factor in program implementation. In many developing countries, rabies control programs are not a high priority on the government’s agenda. The delineation of responsibility and budget allocation between ministries of agriculture and health seems to be unclear [8]. Furthermore, this disease is considered less important in the agriculture sector compared to other livestock diseases of poultry and swine, which have a greater impact on the economy and food security.

A critical component of a successful rabies program is a community that is well-educated on the risk factors associated with, and the control of, rabies [4]. The lack of effective health education programs results in poor awareness of the community on the disease’s situation [9] and incorrect practices towards wound management is likely possible during biting incidents. Most households in rural areas would consult traditional faith healers and apply homemade remedies for bite wounds rather than seeking the help of medical professionals [10]. Whilst not all bites are associated with rabies, people need to be properly educated on the correct first aid to be adopted, such as simple washing of bite wounds with soap, as this can be a decisive factor in preventing rabies deaths [11].

In September 2014, several provinces in Bicol were selected to be pilot areas for an internationally-funded project for rabies elimination. Through the World Organisation for Animal Health (OIE), with funding support from Australia’s Department of Foreign Affairs and Trade (DFAT) Stop Transboundary Animal Diseases and Zoonoses (STANDZ) initiative, together with Philippines’ Department of Agriculture-Bureau of Animal Industry (DA-BAI), an agreement was made to support rabies elimination in two priority areas, Masbate and Albay, as well as to provide vaccination support to Camarines Norte and Camarines Sur. From September 2014 to June 2017, the OIE STANDZ Rabies Project provided technical support for aligning the National Rabies Prevention and Control Program (NRPCP) with international standards, provided vaccines from the OIE Bank, conducted capacity building for new vaccinators, supported rabies information dissemination campaigns, and conducted multi-sectoral meetings, among others.

Rabies awareness in the community is very important in creating effective control measures in the Philippines; however, very little information has been published about this. The conduct of the KAP survey provided additional information to the implementers on which areas of the Rabies program should be improved (knowledge on rabies, vaccination and responsible pet ownership, wound bite management, and prevention and control) based on the pet owners’ response. This study aimed to compare and assess the general knowledge, determine the attitude, and identify the practices of respondents on rabies and its related issues, including biting incidents and RPO, during the pre-implementation (PRI) and post-implementation (POI) stages of the OIE STANDZ Rabies Project in its pilot areas.

## 2. Methods

### 2.1. Study Area

A community-based study was conducted by the OIE STANDZ Rabies Project in pilot areas of Camarines Norte (2320.07 sq. km, 14°8′20.5′′ N 122°45′47.89′′ E), Camarines Sur (5497.03 sq. km, 13.5250° N, 123.3486° E), Albay (2575.77 sq. km, 13.1775° N, 123.5280° E), and Masbate (4151.78 sq. km, 12.3060° N, 123.5589° E). The study was conducted in two periods, namely: April to May 2015 (PRI) and February to April 2017 (POI).

### 2.2. Study Design and Sample Size

**Figure 1:**
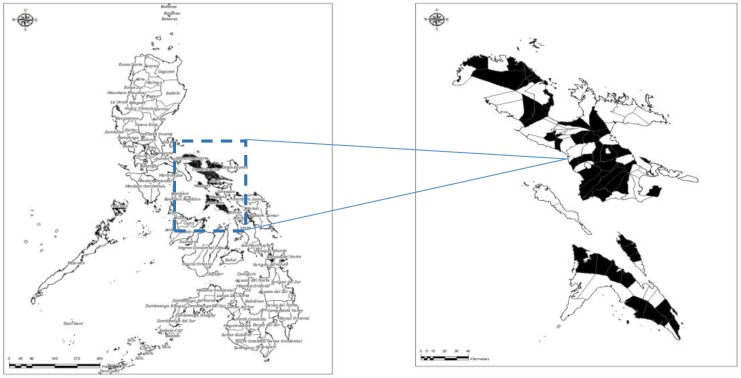
Map showing the four pilot provinces included in the OIE STANDZ Rabies Project. Inset showing the cities/municipalities included in the community awareness survey.

A quantitative descriptive method was employed in this study to determine the knowledge, attitudes, and practices (KAP) of pet owners concerning rabies, including dog bites. In every village in a municipality, respondents were selected, depending on the presence of pets in their household. The same set of municipalities was involved in both PRI and POI studies but not necessarily the same set of pet owners. Municipalities chosen were either 3rd- or 4th-class municipalities. Each district in a province was represented by at least two municipalities. A total of 1088 respondents was included in the PRI study and 1380 in the POI. The study was limited to dog owners of the four provinces aforementioned, since dogs are the primary reservoir host of rabies in the country. Figure 1 shows the map of the four pilot provinces in this study.

### 2.3. Data Collection and Analysis

With coordination from the City Veterinary/Municipal Agriculture Office (CVO/MAO), researchers were accompanied by a staff member from the mentioned offices. Personal interviews were carried out using a structured questionnaire. The questionnaire was developed in English but translated to Tagalog (national language) or *Bikolano* (local dialect) to ensure good comprehension of the questions. One adult in each household was selected to be a respondent in the study. Respondents were briefed before the start of the interview about the purpose of the study. The questionnaire included items regarding the respondent’s profile, vaccination history of their dog/s, the presence of biting incidents and first aid adopted, and other general questions regarding rabies, including its transmission and symptoms. The data were collated in Microsoft Excel and presented using descriptive statistics. Frequency and percentages were used for analysis, including PRI and POI percentages. A statistical analysis using Statistical Analysis System (SAS) was performed to compare the PRI and POI proportions.

## 3. Results

### 3.1. Socio-Demographic Profile of Respondents

Most of the respondents were female (PRI: 57%, POI: 54%), a mother (PRI: 42%, POI: 50%), and married (PRI: 71%, POI: 79%). The majority graduated from at least high school level (PRI: 28%, POI: 23%). Table 1 summarizes the results of the KAP survey of the socio-demographic profile of respondents.

### 3.2. Knowledge on Rabies, Species Affected, and Clinical Signs

Most pet owners had heard about rabies, mostly from radio (PRI: 23%, POI: 11%), health workers (PRI: 19%, POI: 14%), television (PRI: 41%, POI: 19%), neighbors (PRI: 22%, POI: 9.4%), and veterinarians (PRI: 4.5%, POI: 10%). Dogs (PRI: 54%, POI: 62%) and humans (PRI: 67%, POI: 29%) were the usual species affected with rabies according to the respondents. They identified fear of water (PRI: 18%, POI: 20%), aggressiveness (PRI: 16%, POI: 29%), and salivation (PRI: 43%, POI: 24%) as the most common signs of dog rabies. Common signs reported in humans were also fear of water (PRI: 26%, POI: 18%) and salivation (PRI: 22%, POI: 18%). Others admitted that they did not know anything about the signs of rabies in humans (PRI: 29%, POI: 8.9%). Table 2 summarizes the results of the KAP survey on the respondents’ knowledge on rabies.

### 3.3. Transmission of Rabies, Its Prevention, and Presence of Government Rabies Programs

Respondents were asked for their knowledge on how dogs acquire rabies. Common answers were bite of another rabid dog (PRI: 18.1%, POI: 38.0%) and scavenging garbage (PRI: 21.3%, POI: 34.0%). There were also respondents who did not know anything on how rabies is being transmitted (PRI: 30.8%, POI: 13.5%). Others mentioned that dirty food and environment can be reasons why dogs acquire rabies (PRI: 28.3%, POI: 2.9%). On the other hand, rabid dog bite (PRI: 58.7%, POI: 79.5%) and eating dog meat (PRI: 2.4%, POI: 7.0%) are usual responses on how humans get infected with rabies. Similar to dog rabies transmission, there were people who did not know how the virus can be transferred to humans (PRI: 19.3%, POI: 8.1%). Meanwhile, to prevent rabies, pet owners believed that the best measures to prevent rabies in dogs are vaccination (PRI: 56.0%, POI: 92.8%) and confinement of dogs (PRI: 14.8%, POI: 6.1%). Some pet owners also mentioned that proper feeding, regular bathing, and maintaining a clean habitat (PRI: 31.0%, POI: 1.1%) are important rabies preventive measures. A majority of them know that pets should be vaccinated every year (PRI: 41.7%, POI: 92.3%). The data also indicated that the majority had heard about rabies-related local ordinance (PRI: 59.3%, POI: 59.7%) within their municipality (PRI: 63.0%, POI: 90.9%) or a legal mandate regarding RPO (PRI: 37.0%, POI: 9.1%). The majority felt the presence of rabies-related programs (PRI: 69.5%, POI: 96.0%), such as dog vaccination (PRI: 93.2%, POI: 89.9%) and rabies seminars (PRI: 6.1%, POI: 5.4%), during both study periods. Table 3 summarizes the results of the KAP survey on the transmission of rabies, its prevention, and presence of government rabies programs.

### 3.4. Dog Ownership and Attitude towards Biting Incidents

Most of the respondents had at least 1–2 dogs in their household (PRI: 80%, POI: 82%). Totals of 2193 and 2421 dogs were owned by the respondents, with an average of 1.83 and 1.75 dogs per household in the PRI study and the POI study, respectively. Vaccination history of dogs was noted from the respondents. During the PRI period, 58% were vaccinated (1272 of 2193) while in the POI, the history reflected dog vaccination coverage of 37% (890 of 2421) in 2015, 54% (1300 of 2421) in 2016, and 37% (890 of 2421) in 2017. Some of the household members incurred dog bites (PRI: 21%, POI: 21%), where bite victims were usually bitten by their own household dogs (PRI: 52%, POI: 35%) or roaming dogs with an owner (PRI: 34%, POI: 40%). A few first aid measures which were adopted by the bite victims were washing with soap (PRI: 83%, POI: 51%), visiting ‘*tandok’* (PRI: 29%, POI: 22%), and consulting an Animal Bite Treatment Center (ABTC) (PRI: 52%, POI: 39%). Observing the biting dog within the observation period (PRI: 26%, POI: 33%) and the immediate killing of the biting dog (PRI: 8.5%, POI: 12%) were some of the actions taken by bite victims toward the biting dog. Table 4 summarizes the results of the KAP survey on dog ownership and attitude towards biting incidents. 

### 3.5. Attitude towards Suspect Rabid Animal Sightings

Respondents were asked about the actions to be done should they see animals with rabies-like symptoms. The answers included immediate killing of the dog (PRI: 20%, POI: 14%), capturing and observing the dog (PRI: 4.2%, POI: 13.3%), doing nothing (PRI: 14%, POI: 6.0%), and others, such as walking away from the suspect rabid animal (PRI: 36%, POI: 3.9%). A notable increase has been noted in people who responded to report rabid dog sighting to authorities in POI (63%) than PRI (26%). Of the people who answered that they reported to authorities, most of them mentioned that they will report suspect animals to village officials (PRI: 67%, POI: 80%). Table 5 summarizes the results of the KAP survey on the attitude towards suspect rabid animal sightings.

### 3.6. Duties of Owner towards Pets

Pet owners were also asked questions on common practices in taking care of pets. Overall, the most common answers were submitting of dogs for vaccination, confining dogs, and providing dogs with shelter and food. There was improvement (more than twofold) in the response of pet owners to submit their dogs for vaccination, from the PRI to the POI. Table 6 summarizes the results of the KAP survey on the duties of the owners towards pets.

## 4. Discussion

**Figure 2:**
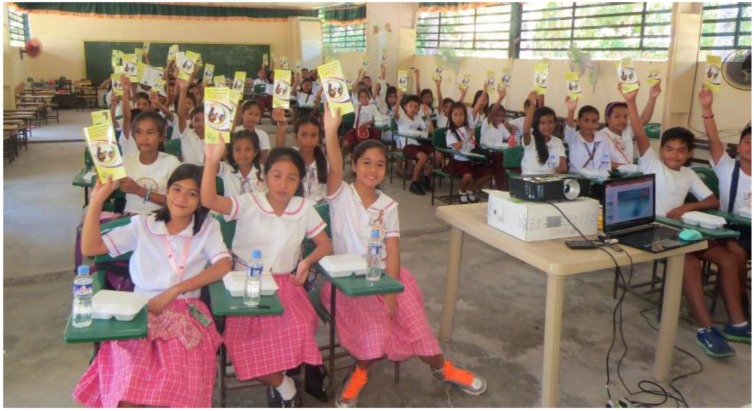
Rabies information dissemination campaign for elementary pupils during Rabies Awareness Month (March) in the province of Masbate.

Community awareness is crucial in rabies prevention and control. Figure 2 shows a rabies-related information dissemination campaign for elementary pupils, which is regularly done during the national celebration of Rabies Awareness Month every March. Therefore, to efficiently increase awareness, the knowledge gap in the community must be identified and targeted [1]. The level of community awareness on different aspects of rabies, including its prevention and control, was investigated in this study. More importantly, the same study was conducted in two different periods with the same set of municipalities included in the sampling frame to evaluate if there was any effect on rabies awareness on pet owners brought about by the almost three-year implementation of the OIE STANDZ Rabies Project across the four provinces in the Bicol Region. 

In many parts of the study, the pet owners’ level of rabies awareness was seen to improve during the post-implementation study. However, there were still some questions which were answered incorrectly; thus, rabies information dissemination must continue at the community level. Females, including mothers, represented the majority of dog owners and respondents for this study. In the Philippines, mothers are the primary care-givers for the children and are thus well-placed to also care for the household dogs [12]. The majority are also high school graduates and have a basic understanding of how to take care of pets and perform activities involving RPO. With the advent of media and technology, many of the pet owners heard news or information about rabies from television; thus, this channel of media is more effective in delivering information to the public. Since most respondents also came from rural areas, radio is also a common source of knowledge. This was true in another study in India where mass media (television/radio/newspaper) was the most common source of information regarding rabies [13]. Veterinarians as a source of knowledge was seen to have a twofold increase in the POI period. This can be accounted to an increase of rabies-related activities conducted in the pilot areas with the support from STANDZ. In both studies, more than half of the respondents believed that humans and dogs are the species affected with rabies. However, during the POI study, there were less than half of the respondents who knew that rabies can also be transmitted to humans. This is a point of concern, since, although they might associate dogs with rabies, they do not understand that a bite or lick on broken skin can also transmit this deadly disease. A considerable number of people did not know that other species (all mammals) can also be affected by rabies. The Anti-Rabies Act of 2007 indicated that rabies concepts must be incorporated into the school curriculum. However, it was only this year (2017) that the move to formally integrate rabies concepts into the school curriculum was done at the national level, through the initiative of the Global Alliance of Rabies Control (GARC) in cooperation with DA-BAI and the Department of Education. This program is estimated to support 21 million students in 46,264 public schools as well as their teachers and parents [14].

Considering all platforms through which respondents have obtained knowledge on rabies, the common clinical signs recognized by pet owners in both dogs and humans were fear of water, aggressiveness, and drooling. Both humans and animals exhibit a fear of water or ‘hydrophobia’ when the animal is trying to drink water due to the spasm of the accessory respiratory muscles of the neck, pharyngeal muscles, and diaphragm followed by extension of the neck and a feeling of dyspnea [15]. When the virus has already reached the salivary glands from the brain, drooling happens due to the paralysis of this organ [16]. Since fear of water, aggressiveness, and drooling are the easiest to be observed and most commonly heard in different media outlets, these clinical signs were noted to be common knowledge from the pet owners. Interestingly, the number of people who did not have any knowledge on the clinical signs in either humans or animals decreased by three- to fourfold in the POI study. This means that increasingly, people are becoming more aware about the disease. Whilst mass media contribute to this, we must also take into account the presence of the intensified rabies campaign present in the pilot areas, which most likely also raised awareness in the community.

The number of pet owners in the POI who knew that dog rabies can be transmitted through a bite doubled compared to the PRI. However, there were still a considerable number of people who believed that scavenging garbage can infect dogs with rabies. This belief could be related to the traditional beliefs about stray dogs that are often observed scavenging. Some people believed that dogs become sick through a dirty environment and eventually become rabid. Others stated that stray dogs are at greater risk to receive a bite from rabid dogs; therefore, this study suggests to program implementers to strictly implement stray dog control to reduce the spread of rabies or other diseases through contact with infected dogs. More people are becoming aware of rabies transmission, with a twofold decrease in people who did not know anything about this. There were a few respondents during both PRI and POI who believed that eating dog meat is a potential source in acquiring rabies. Eating cooked dog meat which is suspected to come from a rabid animal cannot transmit the disease to humans, since the virus is killed by the high temperature of cooking. However, the danger of eating dog meat lies with the person who killed the dog and prepared the meat as contact with the infected saliva via broken skin or mucous membrane, the consumption of uncooked meat and meat preparation are risks for the transmission of rabies [16]. In the Philippines, there are some provinces where people consider raw dog meat, known as ‘*kilawin*’, as a delicacy, especially during alcohol drinking sessions. These people are at risk to exposure to rabies if the dog happens to be infected. Even though the risk of transmission varies, all possible modes of transmission, including through bite, contact with saliva, and consumption of animal products from diseased animals should be avoided [17].

During the POI, an overwhelming response was noted with regard to vaccination being reported as the most important rabies control measure. While vaccination is the most effective measure to control rabies, restricting dogs from roaming freely, bathing dogs, and cleaning their cages were also mentioned. This does not have a direct impact on the transmission of the virus but are components of RPO which should also be improved. Almost all pet owners knew that vaccinations should be carried out every year in the POI period compared to pet owners during the previous study. This could be a direct result of the MDV initiated by most agriculture/veterinary offices. Most respondents were aware of the local ordinances that had been implemented by cities and municipalities within each territorial jurisdiction. There was an increase in the number of pet owners who were aware of the rabies program in their municipality in the POI, compared to the PRI, particularly to dog vaccination.

The number of dogs owned in each household remained almost the same, at 1–2 dogs, in both study periods. Unfortunately, upon checking the vaccination history of the dogs in the POI, out of the 2421 dogs surveyed, there was a 54% (2016) and 36.8% (2017) vaccination coverage, values which are below the 70% vaccination rate recommended for effective control of the disease. Since the data from the POI were only collected in February to April 2017, this might be too early to notice an impact because the MAO/CVO may have only just started its campaign. There may be an increase in awareness observed in many areas during the POI; however, this does not correlate with the number of dogs submitted for vaccination. This result should be considered by program implementers because many of the vaccination reports in the pilot area indicated that the 70% vaccination rate has been reached. MDV might have only reached urban villages but not the remote areas; thus, there is a lower reporting of vaccination in this survey as the study was conducted in both urban and rural villages. Furthermore, there might have been a low turn-out because dogs under the age bracket of 1 year (37%, 897 of 2421 dogs) were included in the POI study. Unfortunately, data on the age bracket was not gathered in the PRI.

MDVs are often performed in a city/municipality over a two-to-three month period depending on its area size. In the Philippines, if few dogs are submitted to a central vaccination site, then house-to-house MDV is also done. If a pet owner has dogs which are not three months of age, they will not yet be vaccinated. Vaccinators advise them to visit the MAO/CVO once the dog reaches the recommended age. However, pet owners, especially in rural areas, will not bother to bring their dogs to the MAO/CVO because of transportation-related expenses and a lack of household members to handle the dogs in these offices. With the presumption that maternal antibody will interfere with the immune response before three months of age, vaccination is not recommended in this age group [18]. However, in studies in Tanzania and South Africa, puppies (under three months of age) responded well to a standard dose of high-quality vaccine [19]. A similar study in Tunisia also yielded a protective antibody titer (>0.5 IU/mL) without evidence of showing maternal antibody interference [20]. Therefore, in rabies-endemic countries, puppies should be considered in vaccination campaigns because delaying vaccination until the 3rd month of age, especially puppies that came from non-vaccinated dams, means that they may not have the chance of being vaccinated at all. Moreover, analysis of the 10-year data from the Regional Animal Disease Diagnostic Laboratory-Bicol Region, showed that 33% of all of the rabies-positive animals are 1 year of age and below, which also includes animals 3 months of age and below. This data shows a higher prevalence of rabies in younger animals, thus the vaccination of puppies should be ensured during MDV.

Some areas in the Bicol region have refined their strategies in the conduct of MDV. Good practices that were documented include the hiring of a community animal health worker (CAHW) or CAHW per village (recommended by their village leader), who would be responsible to conduct an initial census of the dog population and to vaccinate the whole village within a certain period of time dictated by the MAO/CVO. Some CAHWs learned that it was better to vaccinate after office hours and weekends so that owners will be present to handle their pets during vaccination. In other municipalities, socio-civic organizations were tapped as volunteers to augment the needed manpower in MDV. An increase in vaccination accomplishment is achieved through well-informed pet owners willing to cooperate with the government’s rabies-related activities.

At least one bite incident in the household was documented in twenty percent (20%) of the respondents. Usually, pet owners have been bitten by their own dogs. There was an increase in the number of biting dogs that are roaming but are owned; thus, there was no improvement seen in the control of stray dogs in between the two study periods. Most of the bite victims performed washing of the wound with soap, which is one of the most important first aid measures in any bite injury; however, there was a decrease in the number of people performing this during the POI. Consulting ABTCs to seek medical attention was also carried out by bite victims, but a decrease in the number of people doing this was documented in the POI. Consulting ‘*tandok*’ or traditional faith healers and the application of garlic as a topical first aid were still adopted by some bite victims from both study periods. This is not unique to the Philippines, as bite victims from countries such as India and Bangladesh also applied chili oil and turmeric powder as home remedies in their bite wounds [12,21].

Observation of the biting dog was carried out in most biting incidents. There were less people in the POI who did nothing, and an increase in bite victims immediately killing the dog, was noted. In many of the animal rabies cases investigated within the pilot areas, a rabid dog bit more than two people. In worst cases, rabid dogs bit at least ten people. If situations such as these arise, the community comes together to find and kill the aggressive dog and stop the series of biting. In cultures where they are not familiar with dog handling and restraint, the practice of killing is very common [22]. A study in India reported that 43% of the respondents felt that killing stray dogs and suspect aggressive dogs is the best method for controlling rabies [11]. Conversely, in one study, there was no evidence that culling of dogs alone made a significant impact on reducing dog population densities and the number of rabies cases; the confounding factor was the dogs’ high population turnover [21].

There was a significant improvement in the POI with regard to the reporting of rabid dog sightings to authorities, more specifically to village officials. This may be a result of the STANDZ initiative to support community-based orientation on the Anti-Rabies Act. Aside from the human health, animal health, and education counterparts, village officials were also invited to participate in this meeting. In this forum, the roles and responsibilities of each agency towards the implementation of rabies control programs were discussed. Since village officials are considered the frontline in small communities, they must be educated on the importance of their role in preventing and controlling rabies. By underscoring the importance of their position in the program, they will feel empowered and are more likely to effectively perform their job in educating their constituents. Following protocols such as capturing the dog involved, observing them for the next 14 days, and submitting the head sample (if the biting dog dies within 14 days) would be very helpful in preventing the spread of virus from its source. During the PRI, most pet owners replied that they would just walk away from suspect rabid dogs. This reply decreased significantly in the POI, which signifies that pet owners are becoming more conscious of their responsibility as a pet owner to report suspect rabid dogs to authorities.

Pet owners have also recognized their responsibility of submitting their dogs for vaccination. Improvement was realized in the POI study, where more respondents chose this practice as a way to take care of their pets. Meanwhile, almost the same number of people across both studies answered that they should not allow dogs to roam freely. This should prompt the implementing agencies to place more emphasis on education and to create policies that would strengthen the control of stray dogs in the community, since most rabid dogs are strays.

## 5. Conclusions

The Bicol Region has gained substantial knowledge, attitude, and practices on rabies prevention and control and other related issues since the initial PRI study was conducted. Previously, most of the respondents knew very little about rabies and would do nothing upon seeing rabid dogs. After the support given by OIE STANDZ Rabies Project to the pilot provinces, many are now actively reporting sightings of suspect rabid dogs and properly managing dog bite cases. Most respondents have agreed that vaccination is an effective preventive measure to control rabies. However, there are still certain management practices that need to be improved, such as reducing reliance on traditional faith healers for bite wounds, improving rabies vaccination coverage in both rural and urban villages, and controlling stray dogs.

Now that the OIE STANDZ Rabies Project has come to a close, municipalities and cities should institutionalize the implementation of self-sustaining rabies programs, building upon the lessons learned from the project, such as: (1) continuous regular information campaigns directed towards the pet owners (up to the village level) in order to increase the public’s awareness on rabies and related issues; (2) implement ordinances and strictly impose penalties for violators; (3) collaborate with other relevant agencies and organizations, and create strategies to increase MDV coverage in cities and municipalities; (4) design programs to regulate traditional faith healers, and discourage the public from consulting them; (5) involve village officials in the rabies program; and (6) establish a proper referral system for dog bite incidents.

Strategies and tools for a more holistic rabies program implementation are widely available at the international level, yet its operationalization at the community remains as a challenge. Many pet owners actively participated in different information dissemination activities organized by the project. As a result, the knowledge gained by the residents of the community was translated into actions. In the pilot areas, coordination across all sectors (pet owners to village officials, municipal/city animal and human health authorities, and personnel of the animal diagnostic laboratory) has greatly improved during the reporting of those suspect rabid dogs which have eventually turned out to be rabies positive. Behavioral change in the community is reflected by their active response to biting incidents and rabid dog sightings, which coincided with the results in the POI phase. This shows that efforts can effectively be focused first at the community level (which includes empowering village officers) and can serve as a model for implementation at a larger scale as a sound program starts at this level.

Government efforts should likewise be complemented by a cooperative community willing to support activities to promote rabies awareness and responsible pet ownership. A harmonious relationship between the government and its communities and the education of its constituents on this disease will help reduce the incidence of rabies.

## Figures and Tables

**Table 1:** Socio-demographic profile of respondents.

Parameter	Pre-Implementation *N* = 1088 (%)	Post-Implementation *N* = 1380 (%)	*p*-Value
***Profile***			
Father	419 (38.5)	524 (37.8)	1.2777
Mother	453 (41.6)	691 (50.0)	**0.00003**
Son/daughter	178 (16.4)	164 (11.8)	1.9990
Other	38 (3.5)	1 (0.07)	2.0000
***Sex***			
Male	464 (42.6)	624 (45.2)	0.1965
Female	624 (57.3)	756 (54.7)	1.8035
***Civil Status***			
Single	199 (18.3)	157 (11.3)	2.0000
Married	776 (71.3)	1097 (79.4)	**0.000003**
Separated	11 (1.0)	19 (1.3)	0.4995
Widower	67 (6.2)	81 (5.8)	1.3223
Live-in	35 (3.2)	26 (1.8)	1.9739
***Educational Attainment***			
Did not go to school	2 (0.2)	4 (0.2)	1.0000
Elementary Level	69 (6.3)	93 (6.7)	0.6915
Elementary graduate	181 (16.6)	244 (17.6)	0.5151
High school level	155 (14.2)	216 (15.6)	0.3357
High school graduate	305 (28.0)	319 (23.1)	1.9944
Vocational graduate	46 (4.1)	64 (4.6)	0.5516
College level	149 (13.7)	188 (13.6)	1.0571
College graduate	164 (15)	231 (16.7)	0.2545
Postgraduate	17 (1.6)	10 (0.7)	1.9665

**Table 2:** Knowledge on Rabies.

Parameter (Knowledge on Rabies)	Pre-Implementation *N* = 1088 (%)	Post-Implementation *N* = 1380 (%)	*p*-Value
***Sources of Knowledge***			
Newspaper	53 (4.9)	64 (7.5)	**0.0143**
Radio	255 (23.4)	97 (11.4)	2.0000
Relatives	116 (10.7)	42 (4.9)	2.0000
Health workers	205 (18.8)	117 (13.8)	1.9973
Veterinarians	49 (4.5)	87 (10.2)	**0.0000**
TV	489 (44.9)	158 (18.6)	2.0000
Others (seminars, printed materials, barangay officials)	183 (16.8)	161 (19.0)	0.1986
***Species affected with Rabies***			
Humans	732 (67.3)	406 (29.4)	2.0000
Cats	264 (24.3)	260 (19.0)	2.0000
Dogs	587 (53.9)	860 (62.3)	0.0000
Don’t know	83 (7.6)	177 (12.8)	0.0000
Others	100 (9.2)	36 (2.6)	2.0000
***Signs of Rabies in dogs***			
Fear of water	196 (18.0)	277 (20.1)	0.1245
Aggressiveness	174 (16.0)	289 (20.9)	**0.0003**
Paralysis	15 (1.4)	36 (2.6)	0.0135
Convulsion	17 (1.6)	49 (3.6)	**0.0003**
Restlessness/delirium	100 (9.2)	152 (11.0)	0.0840
Salivation/drooling	465 (42.7)	334 (24.2)	2.0000
Loss of appetite	73 (6.7)	72 (5.2)	1.9373
Do not know	235 (21.6)	80 (5.8)	2.0000
Others	372 (34.2)		
***Signs of rabies in humans***			
Fear of water	277 (25.5)	243 (17.6)	2.0000
Aggressiveness	89 (8.2)	224 (16.2)	**0.0000**
Paralysis	28 (2.7)	43 (3.1)	0.4763
Restlessness/delirium	39 (3.6)	279 (20.2)	**0.0000**
Salivation/drooling	236 (21.7)	250 (18.1)	1.9919
Loss of appetite	60 (5.5)	115 (8.3)	**0.0012**
Do not know	320 (29.4)	123 (8.9)	2.0000
Others (crazy, fever, convulsion)	438 (40.3)	201 (14.6)	2.0000

**Table 3:** Knowledge on rabies, its prevention, and presence of government rabies programs.

Parameter (Knowledge)	PRI *N* = 1088 (%)	POI *N* = 1380 (%)	*p*-Value
***How can dogs acquire rabies?***			
Bitten by a rabid dog	217 (19.9)	525 (38.0)	**0.0000**
Eating dog meat	14 (1.3)	106 (7.7)	**0.0000**
Licked on broken skin	29 (2.7)	54 (3.9)	0.0819
Scavenging garbage	256 (23.5)	469 (34.0)	**0.0000**
Do not know	369 (33.9)	186 (13.5)	2.0000
Others (dirty food and environment)	339 (31.2)	40 (2.9)	2.0000
***How can a person acquire rabies?***			
Bitten by a rabid dog	704 (64.7)	1097 (79.5)	**0.0000**
Eating dog meat	52 (4.8)	96 (7.0)	**0.0194**
Licked on broken skin	27 (2.5)	20 (1.4)	1.9578
Scavenging garbage	30 (2.7)	47 (3.4)	0.3095
Do not know	232 (21.3)	112 (8.1)	2.0000
Others (dirty environment)	95 (8.7)	8 (0.6)	2.0000
***How to prevent Rabies?***			
Vaccination	672 (61.8)	1281 (92.8)	**0.0000**
Do not allow dogs to roam freely	178 (16.4)	84 (6.1)	2.0000
Others	372 (34.2)	15 (1.1)	2.0000
Do not know	66 (6.1)		
***Frequency of vaccination (N = 672, N = 1281)***			
Every year	500 (74.4)	1273 (99.4)	**0.0000**
Every 6 months	118 (17.6)	83 (6.0)	2.0000
others	54 (8.0)	24 (1.7)	2.0000
Did you hear of any local ordinance?			
Yes	711 (65.4)	824 (59.7)	1.9963
No	377 (34.6)	556 (40.3)	**0.0037**
***What the local ordinance is all about? (N = 711, N = 824)***			
Local ordinance	448 (63.0)	749 (90.9)	**0.0000**
Responsible pet ownership	148 (37.0)	75 (9.1)	2.0000
***Any presence of Rabies Program of the LGU?***			
Yes	834 (76.7)	1325 (96.0)	**0.0000**
No	254 (23.3)	55 (4.0)	2.0000
***What the program is all about? (N = 834, N = 1325)***			
Dog vaccination	777 (93.2)	1192 (89.9)	1.9916
Rabies seminar	51 (6.1)	71 (5.4)	1.5072
Dog neutering	1 (0.1)	55 (4.2)	**0.0000**
Others	5 (0.6)	7 (0.5)	1.2391

**Table 4:** Dog ownership and attitude towards biting incidents.

Parameter	Pre-Implementation *N* = 1088 (%)	Post-Implementation *N* = 1380 (%)	*p*-Value
***Number of dogs owned***			
1–2	868 (79.8)	1134 (82.2)	0.1304
3–4	183 (16.8)	205 (14.9)	1.8021
5 and above	37 (3.4)	41 (2.9)	1.5192
***Vaccination History (N = 2193, N = 2421)***			
2015	1272 (58.0)	890 (36.8)	2.0000
2016		1300 (54.0)	
2017		890 (36.8)	
***Biting Incidence***			
Yes	225 (20.7)	292 (21.2)	0.7618
No	863 (79.3)	1088 (78.8)	1.2382
***Owner of biting dog***			
Owner’s dog itself	116 (51.6)	102 (35.0)	1.9999
Stray with owner	77 (34.2)	117 (40.1)	0.1544
Stray without owner	50 (22.2)	72 (18.5)	1.6944
Others	5 (2.2)	1 (0.4)	1.9533
***First aid adopted***			
Washed with soap	186 (82.7)	150 (51.4)	2.0000
Applied with garlic	38 (16.9)	24 (8.2)	2.0000
Visited ‘tandok‘	64 (28.6)	63 (21.6)	1.9957
Did nothing	24 (10.5)	12 (4.1)	2.0000
Consulted Animal Bite Treatment Center	116 (51.6)	113 (38.7)	2.0000
Others	20 (8.9)	38 (13.9)	**0.0040**
***Action done to the biting dog (N = 225, N = 292)***			
Observed	58 (25.8)	95 (32.5)	0.0980
Killed instantly	20 (8.9)	34 (11.6)	0.3196
Killed and head brought to laboratory	1 (0.4)	3 (1.0)	0.4401
Did nothing	139 (61.8)	154 (52.7)	1.9616
Others	7 (3.11)	6 (2.0)	1.5758

**Table 5:** Attitude towards suspect rabid animal sightings.

Parameter	PRI *N* = 1088 (%)	POI *N* = 1380 (%)	*p*-Value
***What to do if animal develops symptoms of rabies***			
Immediately kill the dog	220 (20.2)	189 (13.7)	2.0000
Report to authority	286 (26.3)	871 (63.1)	**0.0000**
Capture & observe	46 (4.2)	183 (13.3)	**0.0000**
Do Nothing	147 (13.5)	83 (6.0)	2.0000
Others	389 (35.8)	54 (3.9)	2.0000
***Where to report suspect animals? (N = 286, N = 871)***			
Provincial Vet Office	27 (9.5)	133 (15.3)	**0.0137**
Barangay Officials	192 (67.3)	696 (79.9)	**0.0000**
Police	5 (1.9)	3 (0.3)	1.9954
City/Municipal Agriculture Office	25 (8.6)	28 (3.2)	1.9998
Rural Health Unit	17 (6.0)	8 (0.9)	2.0000
Others	25 (8.6)	3 (0.3)	2.0000

**Table 6:** Duties of pet owners towards pets.

Parameter	Pre-Implementation *N* = 1088 (%)	Post-Implementation *N* = 1380 (%)	*p*-Value
***How should dog be taken care of?***			
Submit dogs for vaccination	379 (34.8)	1083 (78.5)	**0.0000**
Do not allow dogs to roam freely	310 (28.5)	360 (26.1)	1.9463
Register dogs	84 (7.7)	134 (9.7)	**0.0082**
Provide dogs with shelter/food	309 (28.4)	854 (61.9)	0.0000
Others (provide vitamin supplements, bathe regularly)	351 (32.3)	77 (5.6)	2.0000
